# Supplementary material for: Functional architecture of Escherichia coli: new insights provided by a natural decomposition approach
Source: Genome Biol. 2008 Oct 27;9(10):R154. doi: 10.1186/gb-2008-9-10-r154 (PMC2760881; doi:10.1186/gb-2008-9-10-r154)
Supplement: Additional data file 1 — Topological properties of the transcriptional regulatory network of E. coli. [file gb-2008-9-10-r154-S1.pdf]

Topological properties of the transcriptional regulatory network of E. coli (Figure S1)

Number of genes	1692
Number of NDRIs	4293
Average path length	2.68
Network diameter	8
Network density	0.003
Coverage (%)	98.42
Avg. clustering coeff.	0.6

Notes:

Network density is the fraction of node pairs that are connected by an edge.

Coverage is the percentage of node pairs that are connected, at least, by one path.

NDRI means Non Directed Regulatory Interaction, disregarding autoregulation (loops).

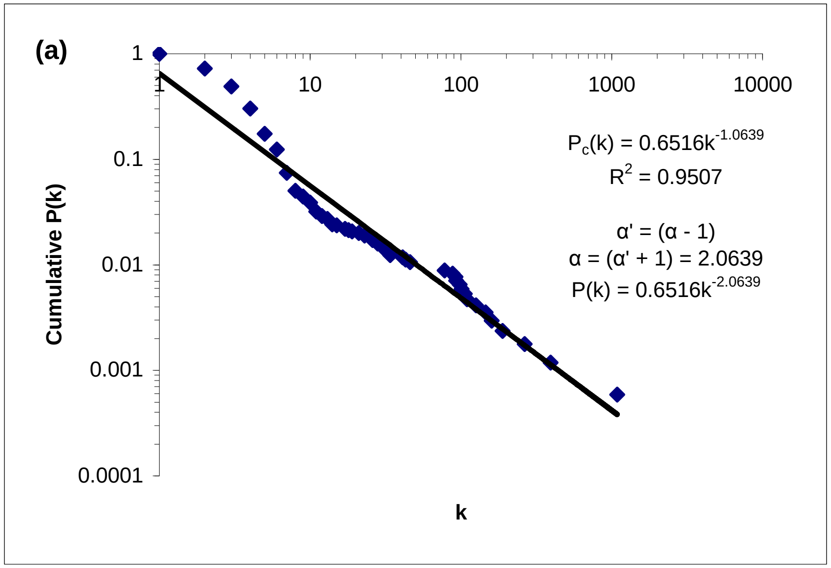

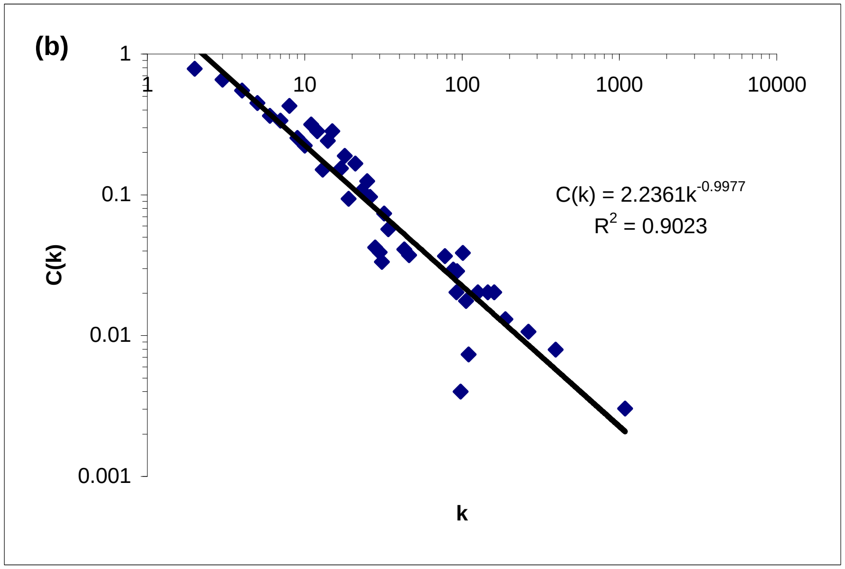

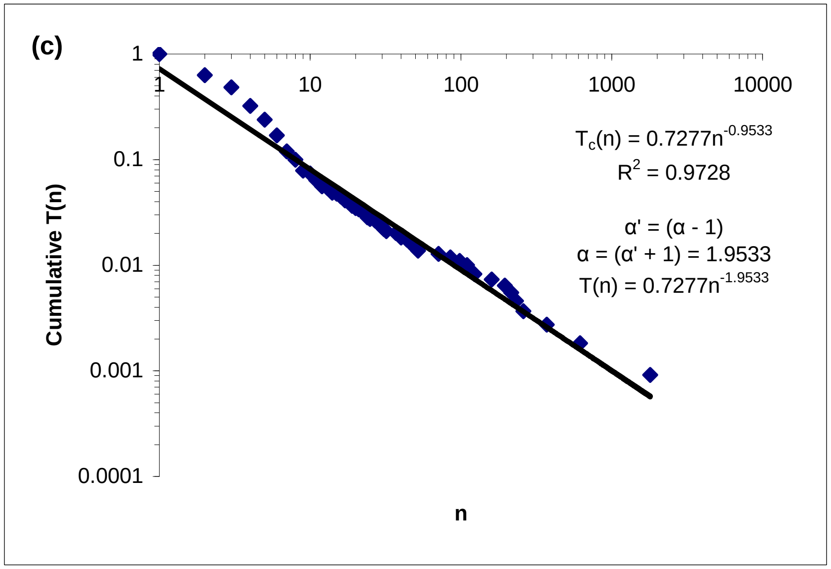

(a) Cumulative connectivity distribution, $P(k)$. Probability that a node has k links. There are only a few hubs connecting a lot of nodes.
(b) Clustering coefficient distribution, $C(k)$. Nodes with only a few links belong to modules, while hubs bridge modules.
(c) Nearest neighbors distribution, $T(n)$. Probability that a node be vertex of n triangles (feedforward, three-nodes feedback, or any flavor of them).
