# Supplementary material for: Functional architecture of Escherichia coli: new insights provided by a natural decomposition approach
Source: Genome Biol. 2008 Oct 27;9(10):R154. doi: 10.1186/gb-2008-9-10-r154 (PMC2760881; doi:10.1186/gb-2008-9-10-r154)
Supplement: Additional data file 2 — Modules identified in this study and their manual and computational annotations. [file gb-2008-9-10-r154-S2.pdf]

Computational and manual annotations of modules (Table S1)

Each module was assigned with a consecutive number as ID. The module 5 is the megamodule, which is subdivided into 39 submodules. Submodules with an 'r' in their ID are simple regulons (26 submodules).

Module	Computational Annotation		Manual Annotation
	**Functional Class according MultiFun**	***p*-value**	
1	Zn	4.96E-09	ATP Binding Cassette High-affinity zinc uptake system.
2	Adaptation to stress	3.55E-07	Zinc homeostasis.
3	Detoxification (xenobiotic metabolism)	3.47E-04	Metal detoxification, P-type ATPase involved in the efflux of Pb(II), Cd(II), Zn(II) and Hg(II).
4	L-ascorbate degradation	8.21E-06	Transport and catabolism of 2,3-diketo-L-gulonate via L-ascorbate and L-xylulose.
5.1	Phosphorous metabolism	4.84E-41	Phosphorous metabolism and DNA replication control
5.2	Drug resistance/sensitivity	7.44E-09	Response to multiple stresses (mechanical, nutritional, oxidative, antibiotics, acid pH). Periplasmic and membrane proteins controled by RpoE sigma factor (pili, curli, biofilm).
5.3	Detoxification (xenobiotic metabolism)	1.12E-08	Response to multiple stresses (antibiotics, organic compounds, mechanical, oxidative, xenobiotics). DNA reparation.
5.4	Trehalose degradation, low osmolarity+	1.23E-03	Trehalose degradation in low osmolarity and magnesium transport.
5.5	Amino sugar conversions	5.33E-09	Amino sugars transport and conversion. Surface antigen biosynthesis (lipopolysaccharides and peptidoglycan).
5.6	Sulfur metabolism	9.71E-23	Transport and catabolism of sulfur and nitrogen sources.
5.7	Maltose	1.45E-09	Transport and catabolism of maltodextrins.
5.8	Methionine	3.93E-20	DL-methionine transport and S-adenosyl-methionine biosynthesis.
5.9	2,5-ketogluconate metabolism; gluconate; L-idonate catabolism	1.49E-07	Transport and catabolism of L-idonate and D-gluconate.
5.10	Methylgalactoside/galactose	2.48E-08	Transport of b-methyl-D-galactoside and D-galactose.
5.11	Gluconeogenesis	7.80E-08	Transport and catabolism of fructose. Gluconeogenesis.
5.12	Anaerobic respiration	2.25E-15	Anaerobic respiration and fermentation. Hidrogenase-4 production.
5.13	Aerobic fatty acid oxidation pathway	6.92E-07	Aerobic fatty acid oxidation pathway.
5.r1	DNA replication	4.81E-04	Modulation of chromosomal supercoiling. DNA replication.
5.r2	Drug resistance/sensitivity	2.25E-02	Antibiotic and xenobiotics resistance.
5.r3	Prophage genes and phage related functions	2.54E-02	Antibiotics sensibility.
5.r4	Starvation response	2.29E-04	Aerobic respiration.
5.r5	Arginine	8.80E-16	L-ornithine and L-arginine biosynthesis.
5.r6	Amino acids	1.12E-02	Acid pH homeostasis by lysine.
5.r7	Not annotated	> 0.05	Transport and catabolism of 4-aminobutyrate.

5.r8	Cu+	3.54E-03	Cu^{+2} homeostasis.
5.r9	Salvage pathways of pyrimidine ribonucleotides	2.09E-05	Pyrimidine ribonucleotides degradation (cytidine). Salvage pathways of pyrimidine deoxyribonucleotides.
5.r10	Nucleotide and nucleoside conversions	1.12E-02	Pyrimidine ribonucleotides degradation and conversion.
5.r11	Not annotated	> 0.05	Molybdopterin biosynthesis. Porins regulation.
5.r12	Fatty acid and phosphatidic acid	4.14E-03	Unsaturated fatty acids biosynthesis.
5.r13	Motility (incl. chemotaxis, energytaxis, aerotaxis, redoxtaxis)	2.69E-38	Flagellum biosynthesis. Motility (incl. chemotaxis, energytaxis, aerotaxis, redoxtaxis).
5.r14	Formyl-tetrahydrofolate biosynthesis	6.50E-03	Formyl-tetrahydrofolate biosynthesis. Positive regulation.
5.r15	Formyl-tetrahydrofolate biosynthesis	6.50E-03	Formyl-tetrahydrofolate biosynthesis. Negative regulation.
5.r16	Protein related	3.14E-05	Fe-S cluster assembly. Alanine biosynthesis.
5.r17	Not annotated	> 0.05	D-glucarate degradation.
5.r18	Oligopeptide	1.12E-11	Molybdate and oligopeptides transport. Peptidoglican mureina recycling.
5.r19	Amino sugar conversions	2.17E-08	Transport and catabolism of N-acetylneuraminic acid.
5.r20	Anaerobic respiration	2.51E-11	Anaerobic respiration and fermentation by nitrates and nitrites.
5.r21	pH response	4.61E-05	Sodium homeostasis (Adaptation to high salinity and alkaline pH).
5.r22	Detoxification (xenobiotic metabolism)	1.89E-02	Detoxification of nitric oxide (NO) under anaerobic conditions.
5.r23	Thioredoxin, glutaredoxin	1.46E-04	Oxidative stress response.
5.r24	DNA recombination	7.68E-03	Carbamoyl phosphate biosynthesis and aminopeptidase A/I regulation.
5.r25	Purine biosynthesis	2.53E-14	Purine and pyrimidine biosynthesis.
5.r26	Cell division	2.45E-04	Cell division regulation by Quorum Sensing.
6	D-xylose	2.48E-08	Transport and catabolism of D-xylose.
7	Xanthosine	1.77E-03	Transport and degradation of xanthosine and salvage pathways of purine.
8	D-glucuronate catabolism	7.25E-09	Transport and catabolism of hexuronates.
9	L-ascorbate degradation	1.43E-08	Transport and catabolism of L-ascorbate.
10	Hexose phosphate	1.18E-03	Transport of hexose 6-phosphates.
11	Tryptophan	3.00E-18	Transport and biosynthesis of aromatic amino acid.
12	Threonine catabolism	5.76E-06	Transport and catabolism of L-threonine/L-serine.
13	Cell killing	4.19E-06	Cell killing by hemolysin E.
14	D-galactarate catabolism; D-glucarate catabolism	3.08E-09	Transport and catabolism of D-galactarate and D-glucarate.
15	RNA related	7.08E-03	RNA modification, RNA 3'-terminal phosphate cyclase.
16	Rhamnose catabolism	2.48E-07	Transport and catabolism of D-rhamnose/L-lyxose.
17	D-ribose	4.41E-11	Transport and catabolism of D-ribose.
18	Flagella	3.92E-04	Quorum sensing of flagella and motility.
19	Prophage genes and phage related functions	1.30E-09	Prophage genes and phage related functions.
20	Propionate degradation	1.24E-07	Propionate degradation.
21	Pyruvate dehydrogenase	6.95E-08	Aerobic-Anaerobic respiration, glyoxylate and L-lactate degradation.

22	Phenylacetic acid degradation	9.80E-32	Phenylacetic acid degradation.
23	Protection; The Telurite-resistance/Dicarboxylate Transporter (TDT) Family; tellurite	3.55E-03	Tellurite resistance.
24	Ni++	5.22E-14	Nickel (II) ABC uptake system.
25	Nicotinamide adenine dinucleotide (NAD)	1.77E-03	NAD biosynthesis and NAD salvage pathway.
26	Mn+/H+	1.18E-03	Uptake of Mn(II).
27	2-O-alpha-mannosyl-D-glycerate degradation	1.24E-09	Transport and catabolism of the osmolyte 2-O-alpha-mannosyl-D-glycerate.
28	3-phenylpropionate and 3-(3-hydroxyphenyl)propionate degradation	9.99E-14	Phenylpropionate and 3-hydroxyphenylpropionate degradation.
29	Carbohydrates/Carbon compounds	2.39E-04	Transport and catabolism of melobiose.
30	Glucose/maltose	1.77E-03	Negative regulation of the maltose regulon.
31	Lysine, diaminopimelate	3.84E-05	Lysine biosynthesis.
32	Sugar	5.03E-08	ATP-dependent sugar transporter.
33	Fimbria, pili, curli	4.72E-03	Regulation of *flhDC* and *fimE* (fimbrial complex) these two regulators are involved in extracytoplasmatic functions (pili, curli, fimbia, biofilm).
34	SOS response	4.78E-39	DNA repair and SOS response.
35	Leucine	1.55E-13	Leucine biosynthesis.
36	Carbohydrates/Carbon compounds	9.14E-04	Transport and degradation of lactose.
37	K+	1.98E-08	ATP-dependent high-affinity potassium transport system.
38	Isoleucine/valine	1.33E-04	Isoleucine and valine biosynthesis.
39	3-phenylpropionate and 3-(3-hydroxyphenyl)propionate degradation	2.41E-11	Phenylpropionate and 3-hydroxyphenylpropionate degradation.
40	Glucitol/sorbitol; The PTS Glucitol (Gut) Family	4.34E-08	Transport and catabolism of glucitol/sorbitol.
41	Glycerol degradation I	4.54E-14	Aerobic-anaerobic respiration, Transport and catabolism of glycerol-3-P.
42	Carbohydrates/Carbon compounds	6.95E-03	Transport and catabolism of glycolate.
43	Galactitol; The PTS Galactitol (Gat) Family	4.34E-08	Transport and catabolism of galactitol.
44	Fucose catabolism	5.14E-10	Transport and catabolism of L-rhamnose and L-fucose.
45	Iron dicitrate	1.83E-13	Ferric citrate outer membrane transporter.
46	Carbohydrates/Carbon compounds	2.39E-04	Evolved β-D-galactosidase, α and β subunits cryptic genes.
47	Amino acids	3.56E-04	Transport and catabolism of D-serine.
48	Unassigned reversible reactions	9.54E-04	Dihydroxyacetone utilization.
49	Fermentation	3.11E-08	Aerobic-anaerobic respiration and fermentation, transport and catabolism of C4-dicarboxylic acid fumarate, orotate, malate and citrate.
50	Cyanate catabolism	4.96E-09	Transport and catabolism of cyanate.
51	Cation	3.55E-03	Copper/silver efflux system.
52	Transcription related	1.13E-02	Cold shock, DNA gyrase type II topoisomerase.
53	Carnitine metabolism	1.43E-14	Transport and catabolism of carnitine.
54	Biotin	3.10E-17	Biotin biosynthesis. Fatty acid biosynthesis initial steps.

55	Betaine biosynthesis	1.98E-08	Transport and biosynthesis of betaine (osmotic pressure resistance).
56	Acetoacetate degradation	1.24E-08	Short chain fatty acid transport and acetoacetate degradation.
57	L-arabinose catabolism	1.04E-07	Transport and catabolism of L-arabinose.
58	allose/ribose	2.48E-08	Transport and catabolism of D-allose.
59	Allantoin assimilation	1.83E-18	Transport and catabolism of allantoin.
60	N-acetylgalactosamine	3.84E-05	Transport and catabolism of N-acetylgalactosamine.
61	Arginine degradation III (agmatinase pathway)	3.54E-03	L-arginine degradation.
62	DNA repair	1.79E-05	DNA repair, O6-methylguanine and O4-methylthymine repair system.

Note: The reader is warned about some orthographic errors in the 'Computational Annotation' column. However, we used 'as is' the MultiFun classification system (see Materials and Methods in the main manuscript). At the date this is written (jan/16, 2008) these errors are still present in the flat file downloadable from [69].
