# Supplementary material for: Functional architecture of Escherichia coli: new insights provided by a natural decomposition approach
Source: Genome Biol. 2008 Oct 27;9(10):R154. doi: 10.1186/gb-2008-9-10-r154 (PMC2760881; doi:10.1186/gb-2008-9-10-r154)
Supplement: Additional data file 3 — Intermodular genes found in this study, their biological descriptions and roles as integrative elements. [file gb-2008-9-10-r154-S3.pdf]

Intermodular genes: Biological functions and their roles as integrative elements (Supporting File 1)

This supporting file lists all the identified intermodular genes. We briefly discuss their biological functions, focusing our attention in how they are regulated and their roles as elements integrating different physiological signals.

Each entry represents a transcription unit (TU). Only one TU is listed when the same set of genes are transcribed from more than one promoter. Each entry of this listing is organized as follows: intermodular genes, known regulatory proteins (disregarding hierarchical transcription factors; they are defined in the main manuscript), and the modules where regulatory proteins were classified.

1. ***carAB*** (PurR-[5.r25] ArgR-[5.r5] PepA-[5.r24]). These genes encode a carbamoyl phosphate synthetase involved in the *de novo* biosynthesis of pyrimidines and arginine (regulated respectively by PurR and ArgR) through the common intermediary carbamoyl-phosphate. PepA is the specific regulator for these genes.

2. ***astCADBE*** (ArgR+[5.r5] NtrC+[5.6]). These genes encode the enzymes involved in the degradation of arginine, a nitrogen-rich amino acid. L-glutamate is produced during degrading, an important compound for nitrogen assimilation. NtrC is the general regulator of the nitrogen assimilation pathway. ArgR upregulates the expression of these genes.

3. ***argD*** (PhoP-[5.4] ArgR-[5.r5]). ArgD is a dual enzyme that takes part in the biosynthesis of lysine and arginine. ArgR upregulates the degradation and downregulates the biosynthesis of arginine. The role of PhoP in the regulation of this gene is still unclear.

4. ***rutABCDEFG*** (NtrC+[5.6] PhoP+[5.4]). These genes encode the enzymes involved in the pyrimidine degradation pathway as a source of nitrogen, which makes NtrC regulation evident. The role of PhoP in the regulation of these genes is still unclear.

5. ***nanC*** (NanR-[5.r19] NagC-[5.5]). *nanC* encodes an N-acetylneuraminic acid (NANA) outer membrane channel. NANA enters in the N-acetyl-D-glucosamine dissimilation pathway. NagC is the general regulator of the N-acetyl-glucosamine degradation pathway. Hence explaining the regulation by NagC. NanR is the specific regulator for the N-acetylneuraminic acid degradation.

6. ***nagA*** (PhoP+[5.4] NagC-[5.5]). *nagA* encodes a subunit of the N-acetylglucosamine-6-phosphate-deacetylase, which is involved in the N-acetyl-glucosamine degradation. Which makes clear the role of NagC in the regulation of these genes. The role of PhoP in the regulation of this gene is still unclear.

7. ***gcvB*** (GcvR-[5.r15] GcvA-[5.r14]). *gcvB* encodes a small RNA, which downregulates both DppA and OppA. These proteins are involved in the di- and oligopeptides transport. In this case, GcvR and GcvA work synergically to regulate this gene. *gcvB* has no functional relationship with other *gcv* genes.

8. ***gcvTHP*** (PurR-[5.r25] GcvA-[5.r14]). These genes encode subunits of the glycine cleavage system, which is involved in formyl-tetrahydrofolate biosynthesis pathway. GTP and glycine are intermediaries in this pathway. PurR inhibits both purine degradation and biosynthesis, GTP in this case. GcvA regulates genes involved in the glycine cleavage system.

9. ***guaBA*** **(**PurR-[5.r25] DnaA-[5.1]). This two genes encode enzymes involved in the *de novo* purine biosynthesis, which convert inosine-5'-phosphate into GMP. Both regulators inhibit purine biosynthesis, GMP in this case. DnaA is an important regulator of DNA replication.

10. ***glyA*** (PurR-[5.r25] MetR+[5.8]). *glyA* encodes a subunit of serine-hydroxymethyltransferase. Serine hydroxymethyltransferase converts serine to glycine, transferring a methyl group to tetrahydrofolate, thus forming 5,10-methylene-tetrahydrofolate (5,10-mTHF). MetR is a dual regulator of amino acid biosynthesis, while PurR regulates both nucleotide degradation and biosynthesis.

11. ***purHD*** (PhoP+[5.4] PurR-[5.r25]). *purHD* encode the enzymes that take part in the first steps of the *de novo* purine biosynthesis. PurR is the general regulator of this pathway. The role of PhoP in the regulation of these genes is still unclear.

12. ***malS*** (MalT+[5.7] PhoP+[5.4]). MalS is a periplasmic enzyme that degrades lineal dextrins of at least three glucose residues producing maltohexoses. MalT regulates the degradation of maltose and other maltohexoses. The role of PhoP in the regulation of this gene is still unclear.

13. ***codBA*** (PurR-[5.r25] Nac+[5.6]). These genes encode enzymes for the cytosine metabolism. Degradation of this nucleotide produces ammonia. PurR is the general regulator of both biosynthesis and degradation of nucleotides. Nac is one of the central regulators involved in nitrogen assimilation.

14. ***galETKM*** (HU-[5.r1] GalS-[5.10] GalR-[5.10]). GalR, GalS and HU form a complex to regulate this operon. The enzymes encoded by the *galETKM* operon take part in the UDP-galactose and colanic acid biosynthesis.

15. ***metBL*** (MetJ-[5.8] PhoP+[5.4]). These genes encode enzymes that take part in methionine, homoserine and threonine biosynthesis. MetJ is a repressor of the amino acid biosynthesis. The role of PhoP in the regulation of these genes is still unclear.

16. ***manXYZ*** (NagC-[5.5] Mlc-[5.7]). These genes encode subunits of the mannose PTS permease, which transport mannose with micromolar affinity. PTS system import different exogenous hexoses (even nitrogenated sugars) into cytoplasm. However, NagC and Mlc are transcription factors that regulate the PTS-dependent transport of mannose, enabling the selective transport of this carbon source.

17. ***gabDTP*** (Nac+[5.6] CsiR-[5.r7]). These genes encode enzymes involved in the γ-aminobutyrate (GABA) degradation pathway. GABA can be used as a nitrogen source, so the nitrogen assimilation control (Nac) regulator regulates the expression of these genes. CsiR is the specific regulator of GABA degradation pathway.

18. ***fadL*** (OmpR-[5.2] FadR-[5.13] PhoP+[5.4]). FadL is a long-chain fatty acid outer membrane transporter. OmpR regulates outer membrane proteins, while FadR is involved in regulation of the fatty acids degradation. The role of PhoP in the regulation of this gene is still unclear.

19. ***micF*** (MarA+[5.3] SoxS+[5.3] HU+[5.r1] OmpR+[5.2] Rob+[5.3]). *micF* RNA is an antisense negative regulator of OmpF abundance. *micF* RNA binds to the *ompF* mRNA inhibiting its translation. OmpF is an outer membrane porin that allows the passage of solutes such as sugars, ions, and amino acids that are smaller than 600 daltons. The transcription factors regulating *micF* expression respond to different stresses such as antibiotics, high or low osmolarity, heat, ethanol, salicylate, paraquat and membrane perturbation. Thus, the regulatory role of MarA, SoxS, OmpR and Rob becomes clear. On the other hand, cells lacking the histone-like protein HU form filaments and have an abnormal number of anucleate cells. The complete absence of HU decreases the steady-state level of *micF* RNA, thus increasing the basal level of the OmpF membrane protein.

20. ***acrAB*** (PhoP-[5.4] MarA+[5.3] SoxS+[5.3] Rob+[5.3] AcrR-[5.r2]). AcrAB and TolC assemble a multidrug efflux transport system that confers resistance to multiple antimicrobial agents, solvents, dyes and detergents. Explaining this fact the regulation by MarA, SoxS and Rob. AcrR is the specific repressor of *acrAB* operon. The role of PhoP in the regulation of these genes is still unclear.

21. ***glnK-amtB*** (NtrC+[5.6] GadX+[5.2]). AmtB is an ammonium antiporter. Disruption of this gene impaired growth on ammonium only under acidic conditions. GlnK regulate the expression of *ntr* genes under conditions of nitrogen starvation. NtrC is the general regulator of the nitrogen assimilation pathway. GadX is one of the central regulators of the glutamate acid resistance (GAD) system.

22. ***gltBDF*** (Nac-[5.6] GadE+[5.2]). *glt* genes encode a glutamate synthase. Glutamate synthase catalyzes the single-step conversion of L-glutamine and α-ketoglutarate into two molecules of L-glutamate. In doing so, it simultaneously operates as the major source of L-glutamate for the cell and as a key step in ammonia assimilation during nitrogen-limited growth. Regulation by Nac and GadE is explained by the fact that GABA (γ-aminobutyrate, a compound involved in acid resistance) is produced from L-glutamate

23. ***purA*** (GadE+[5.2] MarA-[5.3]). PurA is an adenylosuccinate synthetase that catalyzes the first step of the *de novo* synthesis of AMP. Both regulators are involved in stress conditions where biosynthesis of AMP may be needed or not.

24. ***hdeAB*** (MarA-[5.3] TorR+[5.2] GadX+[5.2] GadE+[5.2]). HdeA and HdeB are periplasmic chaperones playing a role in resistance to low pH levels under acidic stress. Hence the regulation by the pH response regulators (GadX and GadE) is explained. Both regulators are required to optimum protection against low pH levels. TorR is a negative regulator of GadX transcription. MarA regulator controls several genes involved in the response to multiple antibiotics.

25. ***fliC*** (FliA+[5.r13] GadE+[5.2]). *fliC* encodes the flagellin, the body of bacterial flagellum. Slightly acidic pH turns on flagellum activity, which explains regulation

by GadE. On the other hand, FliA is the sigma factor responsible for the transcription of flagellar genes.

26. ***cadBA*** (GadX+[5.2] CadC+[5.r6]). *cadBA* genes encode a lysine/cadaverine antiporter (*cadB*) and a lysine decarboxylase (*cadA*). CadA is induced under low pH conditions. CadC regulates the expression of *cadAB* operon under low external pH and lysine abundance. It is thought that both, CadA and CadC, may play an important role in the lysine-dependent acid resistance system 4 (AR4). GadX is an important low pH response regulator.

27. ***osmC*** (NhaR+[5.r21] RcsB+[5.2]). OsmC is an osmotically inducible peroxidase which takes part in the defense against oxidative compounds. RcsB is involved in the activation of colanic acid capsule synthesis (*cps*) and cell division (*ftsZ*) genes. NhaR coregulate the transport of cations to avoid high osmolarity.

28. ***ftsAZ*** (SdiA-[5.r26] RcsB+[5.2]). FtsA and FtsZ make the septum to initiate cell division. SdiA is a quorum-sensing regulator and hence is important to coordinate cell division. RcsB is a two-component regulator member of the LuxR family, being the latest another quorum sensing regulator.

29. ***motAB-cheAW*** (FliA+[5.r13] CpxR-[5.2]). MotAB comprise the stator element of the flagellar motor. CheW is involved in the transmission of sensory signals from the methyl-accepting chemotaxis proteins (MCPs) to the flagellar motors. CheA is responsible for CheY phosphorylation. FliA is the sigma factor responsible for the transcription of flagellar genes. CpxR regulator modulates the expression of genes involved in the response of a large variety of stress conditions, being some of them protein damage, starvation, and high osmolarity. Thus, CpxR negative regulation could avoid erroneous sensing of stress by the cell.

30. ***tsr*** (FliA+[5.r13] CpxR-[5.2]). The *tsr* gene product is one of four methyl-accepting chemotaxis proteins (MCPs). It also responds to a variety of repellents and is thermosensitive. FliA is the sigma factor responsible for the transcription of flagellar genes. CpxR regulator modulates the expression of genes involved in the response of a large variety of stress conditions. Thus, CpxR negative regulation could avoid erroneous sensing of stress by the cell.

31. ***fabA*** (FabR-[5.r12] FadR+[5.13]). *fabA* encodes an essential enzyme involved in unsaturated fatty acid biosynthesis. FabR, a fatty acid biosynthesis regulator, negatively regulates its expression. While FadR, a fatty acid catabolism regulator regulates positively this enzyme.

32. ***fabB*** **(**FabR-[5.r12] FadR+[5.13]). *fabB* encodes an essential enzyme involved in short chain unsaturated fatty acid biosynthesis. FabR, a fatty acid biosynthesis regulator, negatively regulates its expression. While FadR, a fatty acid catabolism regulator regulates positively this enzyme.

33. ***ptsHI-crr*** (FruR-[5.11] Mlc-[5.7]). These genes encode enzymes for the glucose-specific PTS transport system. Both regulators downregulate the activity of the transporter when no glucose is present (explaining regulation by FruR) and during heat shock (explaining regulation by Mlc).

34. ***aceBAK*** (FruR+[5.11] IclR-[5.13]). This operon encodes enzymes involved in the glyoxylate shunt. Both regulators maintain the balance between the production of energy (explaining regulation by IclR) and the biosynthesis of building blocks (explaining regulation by FruR) when bacteria are growing in acetate or other fatty acids as sole source of carbon and energy.

35. ***nupC*** (CytR-[5.r9] Nac+[5.6]). NupC is a high affinity nucleoside transporter. CytR regulates nucleoside degradation. Nac is involved in the nitrogen assimilation.

36. ***slp*** (MarA-[5.3] AlpA+[5.r3]). *slp* encodes the starvation lipoprotein involved in the resistance to low pH levels. MarA regulates this gene during entry to stationary phase, while AlpA is the specific regulator of this gene.

37. ***ompC*** (EnvY+[5.r11] CpxR+[5.2] OmpR+[5.2]). OmpC is an outer membrane porin that allows the passage of ions and other hydrophilic solutes which tent to be smaller than 500 daltons. EnvY, CpxR and OmpR sense stresses in the outer membrane modulating the expression of *ompC* as it is needed. EnvY responds to molybdate. OmpR is the general regulator of outer membrane proteins.

38. ***ompF*** (EnvY+[5.r11] CpxR-[5.2] OmpR+/-[5.2]). OmpF allows the passage of solutes such as sugars, ions, and amino acids that are smaller than 600 daltons. EnvY (which responds to molybdate), CpxR and OmpR are regulators involved in outer membrane stress sensing, the location where OmpF may be needed.

39. ***ppiA*** (CytR-[5.r9] CpxR+[5.2]). *ppiA* encodes a peptidyl-prolyl cis-trans-isomerase (PPIase). This enzyme catalyzes the conformational isomerization of prolyl residues in peptides. This facilitates appropriate protein folding, an activity regulated by CpxR. CytR downregulates transport and utilization of nucleosides, which makes unclear its role here.

40. ***ahpCF*** (OxyR+[5.r23] MetJ-[5.8]). These genes encode an alkylhydroperoxide reductase that converts alkylhydroperoxides into their corresponding alcohols using NADH or NADPH. OxyR regulates genes involved in oxidative stress. MetJ is a regulator controlling several genes involved in methionine biosynthesis. Alkylhydroperoxide reductase is induced by sulfate starvation. Methionine is an amino acid that has sulfur in its structure. So it is possible that MetJ acts indirectly like a sensor of sulfate levels in the medium.

41. ***eda*** (GntR-[5.9] KdgR-[5.r17] FruR-[5.11] PhoB-[5.1]). Eda is an oxaloacetate decarboxylase from the Entner-Doudoroff pathway. It is involved in the utilization of gluconate. GntR and FruR repress gluconate utilization thus allowing gluconeogenesis. KdgR controls the degradation of acid sugars through this pathway. The role of PhoP in the regulation of this gene is still unclear.

42. ***edd-eda*** (GntR-[5.9] FruR-[5.11]). Edd is a phosphogluconate dehydratase from the Entner-Doudoroff pathway. *eda* also could be repressed from an internal promoter by KdgR and PhoB as was described previously. Both regulators, GntR and FruR, repress gluconate utilization thus allowing gluconeogenesis.

43. ***nirBDC-cysG*** (FruR-[5.11] NarP+[5.r20]). *nir* genes encode a nitrite-reductase present in high concentrations in the cell. The metabolic role of this enzyme is detoxification, reducing nitrites into ammonia. *cysG* gene product is necessary to synthesize the siro-hemo nitrite-reductase prosthetic group. NarP is an anareobiosis regulator that responds to nitrites. FruR is a catabolism regulator that regulates the expression of *nir* genes under the presence of glucose.

44. ***sufABCDSE*** **(**OxyR+[5.r23] IscR+[5.r16]). *sufABCFSE* genes encode the components of a secondary pathway involved in the assembly of iron-sulfur clusters. IscR is the regulator of the primary pathway involved in the same function. OxyR regulates genes that respond to oxidative stress, where proteins with iron-sulfur clusters are needed.

45. ***napFDAGHBC*** (IscR-[5.r16]). *nap* genes encode a nitrate-reductase. IscR regulates the iron-sulfur cluster biosynthesis, a nitrate-reductase cofactor.

46. ***napFDAGHBC-ccmABCDEFGH*** (NarP+[5.r20] ModE+[5.r18]). *nap* genes could be transcribed from other promoter as was described previously. *ccm* genes encode the cytochrome c biogenesis system, which is only synthesized under anaerobic growth conditions. Nitrate-reductase is a multimeric enzyme that requires molybdenum as a cofactor, explaining the regulation by ModE. NarP responds to nitrate as electron acceptor during anaerobiosis.

47. ***moaABCDE*** (CueR-[5.r8] ModE+[5.r18] EnvY+[5.r11]). These genes participate in the biosynthesis of molybdopterin guanine dinucleotide. This is important for those enzymes that use molybdenum as cofactor. Thus explaining the regulation by ModE. CueR is the copper export regulator, playing a role in the copper osmolarity control in response to the interaction with copper or gold. CueR regulation is still unclear. However a possible link could be the fact that *moa* operon is controlled by the availability of various metals in the environment. EnvY is a regulator that responds to molybdate.

48. ***deoCABD*** (ModE-[5.r18] DeoR-[5.r10] CytR-[5.r9]). These genes encode enzymes for the pyrimidine deoxyribonucleosides degradation pathway. DeoR is the general regulator of the pyrimidine nucleotides metabolism, and CytR takes part in the nucleoside degradation pathway. ModE regulation is still unclear. However, ModE regulation suggests the possible existence of a molybdoenzyme participating in purine/pyrimidine turnover but yet not described in *E. coli*.

49. ***hycABCDEFGHI*** (FhlA+[5.12] ModE+[5.r18]). Genes in this operon encode for the transcriptional regulator HycA (*hycA*) (which activate the transcription of FhlA), the hydrogenase-3 maduration protease (*hycI*), and the hydrogenase-3 (*hycBCDEFGH*) (which is a component of the formate hydrogenlyase complex). This complex uses molybdenum as cofactor, thus explaining the regulation by ModE. FhlA is the transcriptional activator controlling the expression of all formate hydrogenlyases.

50. ***fdhF*** (NarP+[5.r20] FhlA+[5.12]). This gene encodes for a formate hydrogenlyase. NarP regulates genes involved in fermentation or anaerobiosis. FhlA is the transcriptional regulator of the formate hydrogenlyase when molybdate is absent.

51. ***nupG*** (DeoR-[5.r10] CytR-[5.r9]). NupG participates in the transport of ribo- and deoxyribo-nucleosides through the outer membrane. DeoR regulates pyrimidines metabolism, while CytR controls the nucleoside degradation pathway.

52. ***tsx*** (DeoR-[5.r10] CytR+/-[5.r9]). Tsx is a protein involved with the transport of ribo- and deoxyribo-nucleosides across the outer membrane. DeoR regulates pyrimidines metabolism, while CytR controls the nucleoside degradation pathway.

53. ***yhgI*** (GntR-[5.9] IscR-[5.r16]). The *yhgI* gene product is required for utilization of DNA as the sole source of carbon and energy, as well for gluconate transport. The enzyme contains an iron-sulfur cluster, explaining the regulation by IscR. GntR is the specific regulator of this gene.

54. ***hyaABCDEF*** (AppY+[5.r4] IscR-[5.r16]). Genes in this operon encode for the hydrogenase 1. The enzyme contains an iron-sulfur cluster, explaining the regulation by IscR. AppY regulator affects the synthesis of many proteins in a growth-phase-dependent fashion.

55. ***norVW*** (NorR+[5.r22] NarP-[5.r20]). *norVW* genes encode flavorubredoxins that take part in the nitric oxide (NO) reduction. NarP responds to NO as electron acceptor during anaerobic respiration. NorR is the specific regulator of this operon.
